# Spatial Differentiation and Influencing Factors of Water Pollution-Intensive Industries in the Yellow River Basin, China

**DOI:** 10.3390/ijerph19010497

**Published:** 2022-01-03

**Authors:** Haibo Du, Xuepeng Ji, Xiaowei Chuai

**Affiliations:** 1 College of Geography and Environmental Science, Northwest Normal University, Lanzhou 730070, China; 2019212413@nwnu.edu.cn; 2 School of Geographic and Oceanographic Sciences, Nanjing University, Nanjing 210023, China

**Keywords:** water pollution-intensive industries, water pollution-intensive index, spatial differentiation, influencing factors, the Yellow River basin

## Abstract

The structure adjustment and layout optimization of water pollution-intensive industries (WPIIs) are crucial to the health and sustainable development of the watershed life community. Based on micro-detailed data of Chinese industrial enterprises from 2003 to 2013, we analyzed and revealed the spatial differentiation characteristics and influencing factors of WPIIs in the Yellow River Basin (YRB) from 2003 to 2013 by constructing a water pollution-intensive index and integrating kernel density estimation and geographically weighted regression models from a watershed perspective. The results show that: (1) the scale of WPIIs in the YRB showed a growth trend from 2003 to 2013, and the output value increased from 442.5 billion yuan in 2003 to 6192.4 billion yuan in 2013, an increase of 13 times. (2) WPIIs are generally distributed in an east-west direction, and their spatial distribution is river-side, with intensive distribution in the downstream areas and important tributaries such as Fen River and Wei River. (3) WPIIs are generally clustered in high density downstream, but the spatial clustering characteristics of different industries varied significantly. The chemical industries, paper industries, etc. were mainly concentrated in downstream areas. Processing of food from agricultural products was distributed in the upper, middle and downstream areas. Resource-intensive industries such as coal and oil were concentrated in energy-rich midstream areas. (4) Natural resource endowment was the main factor affecting the distribution of WPIIs in the midstream and upstream areas of the basin, and technological innovation played a significant role in the distribution of downstream industries. The level of economic development and industrial historical foundation promoted the geographical concentration of industries. The scale of wastewater discharge and the proximity of rivers influenced the concentration of industries in the midstream and downstream.

## 1. Introduction

Large river basins are the cradle of the development of human civilization, and river systems and their organically connected river basin life systems continue to provide natural resources, inland navigation, ecological environment and many other functions and services for human survival and development [1]. With China’s rapid industrialization process and rough industrial development model, a large amount of industrial wastewater and toxic pollutants are illegally discharged, dumped, and disposed of into water bodies, which has led to the continuous deterioration of the water environment and frequent water pollution incidents, such as the Songhua River Water Pollution Incident in 2005, the Taihu Lake Cyanobacteria Pollution Incident in 2007, and the 4.11 Lanzhou Tap Water Exceeding Standards of Benzene Incident in 2014. Water pollution is a serious threat to China’s water resources security and people’s health [2,3]. As an important point source of pollution, the distribution of various pollution-intensive industries (PIIs) along the river, especially water pollution-intensive industries (WPIIs) greatly hinder the healthy and sustainable development of the life community in the basin.

The Yellow River Basin (YRB) is an important energy, chemical, raw material, and basic industrial base in China, it has undertaken a large number of high energy-consuming and high-polluting industries and become an important agglomeration of PIIs [4,5]. In recent years, contradictions such as the high load of resources and environment in the YRB, shortage of water resources, and water environment problems have become prominent [6]. The 2018 China Ecological Environment Status Bulletin shows that in the Yellow River 137 water quality sections, the proportion of poor V water reached 12.4%, significantly higher than the national average level of 6.7% [7]. Therefore, exploring the spatial differentiation characteristics and mechanism of WPIIs from the perspective of watersheds is of great significance for protecting the water environment, improving the effectiveness of environmental regulations, and promoting ecological protection and high-quality development in the watershed.

Domestic and foreign research on industrial development and its environmental pollution mainly focuses on the pollutant emission pattern of industry or on the manufacturing industry and its environmental effects [8,9,10], the relationship between the transfer of polluting industries and the environmental Kuznets curve [11], or the geographical distribution of PIIs and the agglomeration and transfer of industries [12,13,14]. There are also the definition and identification of PIIs according to different environmental factors, such as the identification and analysis of WPIIs [15,16]. In addition, there are related studies on typical PIIs such as the petrochemical industry and the textile industry [17,18,19].

Relevant scholars focused on analyzing the influence mechanism of PIIs. People have proposed a series of theories to explain the motives and patterns of the industrial layout. The factor endowment theory and law of comparative advantage emphasize that labor, natural resource endowments, capital and technology have important impacts on industrial location, which determines the direction of national and regional specialization [20,21]. The new economic geography theory points out that industrial geographic concentration is determined by the interaction of economies of scale and transportation costs. It is believed that agglomeration can promote knowledge spillover and technological exchanges between industries. While upstream and downstream input-output linkages and equipment supply between industries reduce transportation costs can promote the geographic agglomeration of industries [22]. With the prominence of environmental problems, the impact of policy factors and environmental regulations on the layout of polluting enterprises has received widespread attention [23]. The pollution haven hypothesis suggests that PIIs will shift to underdeveloped areas with lower environmental regulations, and these underdeveloped areas will become refuges for high-polluting industries [24]. The Porter hypothesis suggests that appropriate environmental regulations can promote technological innovations in enterprises, and these innovations will improve the competitiveness of enterprises, thereby offsetting the costs caused by environmental protection [25]. Based on the above basic theories, relevant scholars have studied and concluded that the spatial distribution of PIIs are mainly affected by factors such as resource endowment, economic development level, market and transportation, and environmental regulations [5,26,27]. In addition, scholars have also found that under the background of environmental decentralization, there is a phenomenon of transboundary pollution between the upstream and downstream of the river basin [28,29]. That is, the pollutant discharge at the boundary of the basin is more intensive, and the pollutants will eventually flow to the downstream area through the carrying capacity of the river, resulting in more serious pollution at the boundary or downstream area.

The above studies provide us with a good theoretical basis and empirical examples, but there are still several shortcomings: At present, studies on WPIIs are still rare, and the industrial point source pollution, which is difficult to depict in detail, cannot provide strong support for water pollution prevention and control in areas with prominent water problems. In addition, the watershed is an organic life system formed by a series of rivers, the upstream is connected to the downstream, the left bank influences the right bank, and the tributaries act as the main stream. But the existing studies seldom consider WPIIs into the watershed system, which makes it difficult to reveal the linkage between the development and protection of the industry watershed connected by the river system. Therefore, this study mainly discusses the following: (1) by constructing water pollution-intensive index through industrial wastewater discharged (IWW), chemical oxygen demand discharged (COD) and ammonia nitrogen discharged (NH_3_-N) and the scale-intensity method, we can quantitatively identify WPIIs. (2) Taking the YRB as an example, the spatial differentiation characteristics of WPIIs in the YRB from 2003 to 2013 were analyzed and revealed by fully exploring and correlating the industrial characteristics and basin features from a basin perspective. (3) We revealed how different factors can have positive or negative effects on WPIIs in different regions of the basin. Our results can provide a reference for quantitatively identifying WPIIs and exploring the heterogeneity of their influencing factors.

## 2. Materials and Methods

### 2.1. Study Area

**Figure 1:**
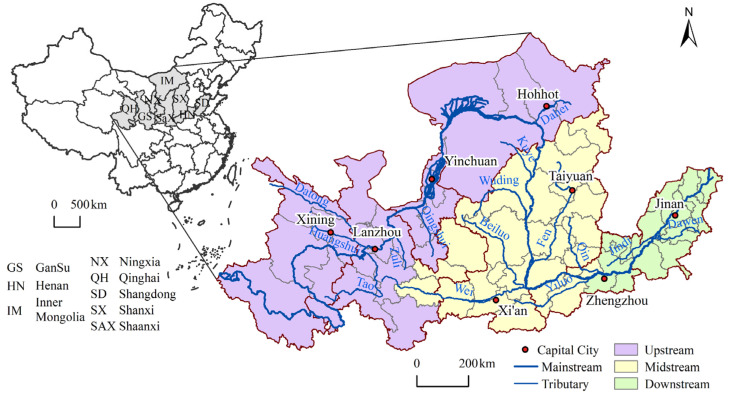
Study area of the Yellow River Basin.

To facilitate analysis and comprehensively consider the accuracy of the study unit and the direct correlation between the distribution of WPIIs and the Yellow River, 66 prefecture-level administrative units flowing through the main stream of the Yellow River were selected as the study objects, involving eight provinces of Qinghai, Gansu, Ningxia, Inner Mongolia, Shanxi, Shaanxi, Henan and Shandong (Figure 1). The land area of YRB is about 1.17 million km^2^, accounting for 12.16% of the national total land area. The terrain is high in the west and low in the east, straddling the three major steps of China, including the Qinghai-Tibet Plateau, the Inner Mongolia Plateau, the Loess Plateau and the North China Plain, with significant differences in natural geography. As of 2018, the GDP of the 66 cities in the YRB was 12.99 trillion yuan, accounted for 14.43% of the national total, and secondary industry accounted for 45.84% of the GDP [30]. The Yellow River is an important water source in Northwest and North China, with poor water resource endowment and uneven spatial distribution of water resources, and the water environment problems are prominent. The per capita share of water resources in the Yellow River are only 27% of the national average, and the exploitation rate of water resources is as high as 80%, far exceeding the 40% ecological warning line of the general basin [31]. The main stream of the Yellow River is slightly polluted, and the main tributaries are seriously polluted. Among the 106 sections, the inferior V category accounts for 16%, and COD and NH_3_-N are the main pollutants.

### 2.2. Data Sources and Processing

The micro data of enterprises came from the Database of Chinese Industrial Enterprises (2003–2013), and the sample scope was all state-owned and non-state-owned enterprises above designated size. First of all, according to the comprehensive measurement results of the WPPI, the industries with a comprehensive index greater than 0.2, according to the Industrial Classification for National Economic Activities (GB/T 4754-2011), the large industry category codes and names of 22 (manufacture of paper and paper products), 26 (manufacture of raw chemical materials and chemical products), 17 (manufacture of textile), 13 (processing of food from agricultural products), 28 (manufacture of chemical fibers), 15 (manufacture of wine, drinks and refined tea), 06 (mining and washing of coal), 25 (processing of petroleum, coking, processing of nuclear fuel), 14 (manufacture of foods) were selected as the research category of WPIIs in this paper. Note that, except for 06 (mining and washing of coal) belonging to the “category B” mining industry, the rest are all “category C” manufacturing industries. Secondly, the above data were pre-processed through various methods, such as filtering, deduplication, repair, and elimination. Finally, geocoding was performed based on the detailed address information of WPIIs, the latitude and longitude information of WPIIs from 2003 to 2013 was obtained and spatially processed, and the WGS_84 geographic coordinate system was converted to the WGS_84_Albers projection coordinate system. The indicator data of the influencing factors came from the China City Statistical Yearbook, while some missing data came from the statistical yearbooks of various provinces and cities, and the statistical bulletin of national economic and social development.

### 2.3. Methods

#### 2.3.1. Water Pollution-Intensive Index

PIIs are industries that produce large amounts of pollutants directly or indirectly during the production process without adequate treatment. The current academic research on the definition of PIIs mainly includes three methods: the pollutant emission scale, pollutant emission intensity, and pollution control cost methods [5,32]. WPIIs refer to industries that cause serious pollution of water resources and the water environment by pollutant discharge, and have specific pollution characteristics and environmental effects associated with water. This paper focuses on industrial point source pollution that has a serious impact on the water environment and water pollution events. Here, WPIIs was identified by constructing a water pollution-intensive index involving scale-intensity [33], and three water pollution indicators, namely industrial wastewater discharged (IWW), chemical oxygen demand discharged (COD), and ammonia nitrogen discharged (NH_3_-N) were selected to participate in the calculation. The above data were obtained from China Environment Statistical Yearbook (2014). The formula used were as follows:

(1) $$I=\frac{1}{3}{\sum_{i=1}^{n}\left(E_{i}\times P_{i}\right)}^{\frac{1}{2}}$$

(2) $$E_{i}=\frac{E_{i}-\min\left(E_{i}\right)}{\max\left(E_{i}\right)-\min\left(E_{i}\right)}$$

(3) $$P_{i}=\frac{P_{i}-\min\left(P_{i}\right)}{\max\left(P_{i}\right)-\min\left(P_{i}\right)}$$

where *I* is the water pollution-intensive index; *E_i_* is the scale of the *i* pollutant discharge of each industry, i.e., “the *i* pollutant discharge of a certain industry/the *i* pollutant discharge of all industries”; *P_i_* is the *i* pollutant discharge intensity of each industry, i.e., “the *i* pollutant discharge of a certain industry/the industrial sales value of that industry”; *E_i_* and *P_i_* are normalized. Through the geometric averaging treatment of the scale and intensity of the three pollutants discharge, and then averaging, the water pollution intensive index of each industry can be obtained. The results are shown in Table 1.

#### 2.3.2. Standard Deviation Ellipse

The standard deviation ellipse is a common method for analyzing the spatial distribution characteristics of point-like geographical elements, which can accurately reveal the spatial distribution patterns of WPIIs in terms of orientation characteristics, dispersion degree and distribution range. Among the basic parameters, the center point represents the central position of industrial layout, the azimuth angle represents the main trend direction of industrial distribution, and the long and short semi-axes represent the direction of industrial distribution and its spatial distribution range, respectively [34].

#### 2.3.3. Kernel Density Estimation

Kernel density estimation is a non-parametric estimation method. It is used to calculate the spatial smoothing estimation value of the feature point and its density in the neighborhood. Through the visual form to characterize the spatial distribution pattern of geographic elements in the study area, it can effectively measure the spatial concentration of WPIIs. The estimated value of the kernel density reflects the density of enterprises, and the larger the value, the denser the distribution of enterprises. The kernel density distribution function was as follows [35]:

(4) $$f(x)=\frac{1}{nh}\sum_{i=1}^{n}k(\frac{x-x_{i}}{h})$$

where: *n* is the number of enterprise points in the neighborhood, *h* is the bandwidth, *k* () is the kernel function, and (*x* − *x_i_*) is the distance from the estimated value *x* to the enterprise point *x_i_*. After several trials, the final output cell was 2.5 km × 2.5 km, and the search radius was 15 km.

#### 2.3.4. Geographically Weighted Regression Model (GWR)

The traditional regression model is mainly based on the least square method to estimate the parameters average or global. The geographically weighted regression model (GWR) extends the traditional linear regression model. Its regression coefficient is no longer a global unified single value, but the spatial location is included in the regression parameters, which can reflect the influence of factors on the distribution of WPIIs that vary with the spatial location, and it is used to explore the spatial non-stationarity and spatial difference of regression coefficients [36]. The essence of GWR is local regression, which is solved by the local weighted least squares method. The model form is:

(5) $$y_{i}=\beta_{0}\left(u_{i},v_{i}\right)+\sum {}_{k}\beta_{k}\left(u_{i},v_{i}\right)x_{ik}+\varepsilon_{i}$$

where: *y_i_* is the dependent variable at point *i*; *x_ik_* is the value of the *k*th independent variable at the *i*-th point; $\left(u_{i},v_{i}\right)$ is the spatial coordinate of the *i*-th sample; $\beta_{k}$$\left(u_{i},v_{i}\right)$ is the local regression coefficient of the *k*th variable at point *i*; and $\varepsilon_{i}$ is the residual.

## 3. Results

### 3.1. Spatial Differentiation of WPIIs

#### 3.1.1. Scale Characteristics

**Figure 2:**
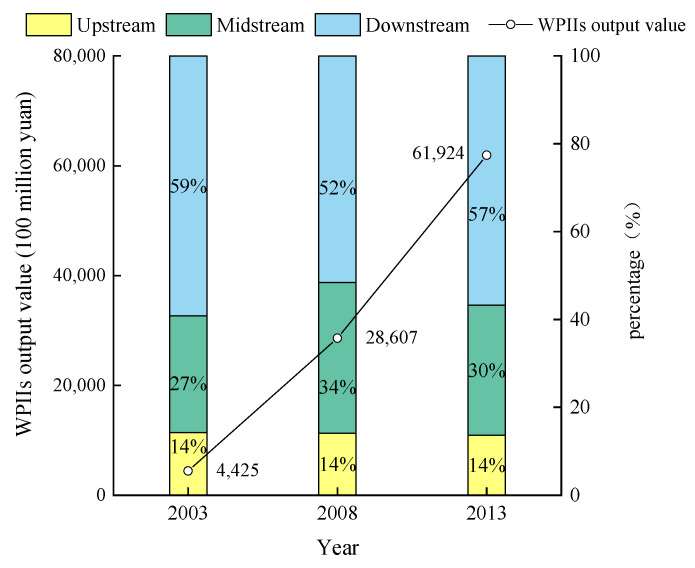
Output value of WPIIs.

The scale of WPIIs in the YRB showed a growth trend from 2003 to 2013, with the output value increasing from 442.5 billion yuan in 2003 to 6192.4 billion yuan in 2013, an increase of 13 times (Figure 2). Within the basin, the share of WPIIs in the upper, middle and lower reaches of the basin was significantly different. The upstream basically remained at 14%, the midstream accounted for about 30%, and the downstream reached more than 50%. The industrial distribution was extremely uneven.

**Figure 3:**
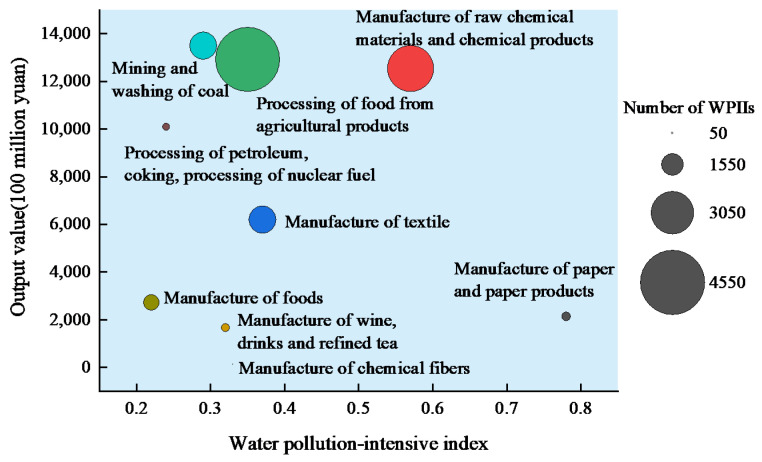
Output value and water pollution-intensive index of WPIIs by industry in 2013.

The statistics of the water pollution-intensive index, output value and number of WPIIs in 2013 are shown in Figure 3. The higher output value contribution industries were mining and washing of coal, processing of petroleum, coking, processing of nuclear fuel and manufacture of raw chemical materials and chemical products. These industries have higher output values and a smaller number of enterprises. Processing of food from agricultural products had a higher contribution to the output value, and the number of enterprises was the largest. The number of enterprises in the textile and manufacture of paper and paper products was relatively small, and the contribution of output value was relatively low, but the pollution of the water environment was more serious. The number and output value contribution of manufacture of chemical fibers, manufacture of wine, drinks and refined tea and manufacture of foods were relatively low.

#### 3.1.2. Directional Distribution Characteristics

**Figure 4:**
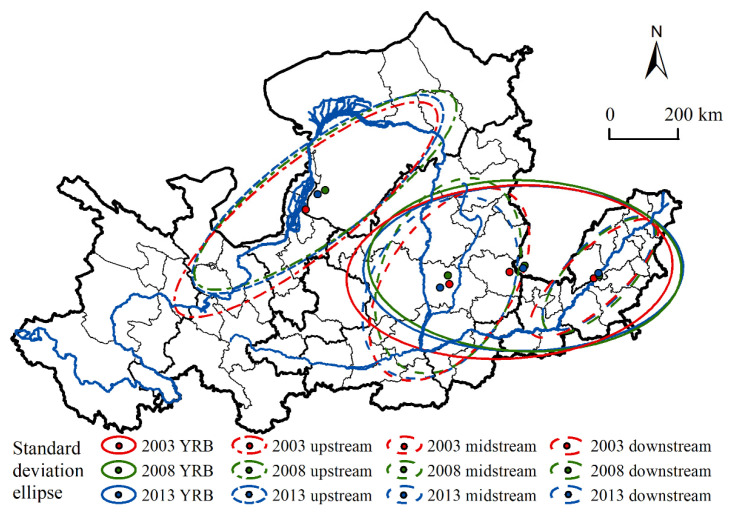
Standard deviational ellipse of WPIIs in the Yellow River Basin.

All WPIIs in the YRB show a significant east-west spatial distribution pattern from 2003 to 2013 (Figure 4). The distribution center was basically located in the boundary between the middle and lower reaches, and the one-time standard deviation ellipse mainly covered the middle and eastern regions of the middle reaches and the lower reaches. During the study period, the long semi-axis decreased and the industries were clustered along the east-west direction. Overall, the WPIIs were clustered in the eastern and downstream areas of the middle reaches of the basin.

Within the basin, the distribution directions of WPIIs in the upper, middle and lower reaches of the basin from 2003 to 2013 were similar, with an overall northeast-southwest direction (in the direction of the main tributaries of the Yellow River) and a symmetrical distribution along the river. In the upstream, the central axis of the industry was in the line of Lanzhou-Yinchuan-Hohhot, and the distribution center shifted from the junction of Yinchuan and Ordos to the northeast to the territory of Ordos. The coverage area moved to the northeast and the WPIIs were clustered along the northeast-southwest direction. In the midstream, the industrial axis was in the line of Taiyuan-Linfen-Xi’an, along the Fen River, and the distribution center was always located in Linfen. The WPIIs were clustered in the northeast-southwest direction. In the downstream, the WPIIs were symmetrically distributed in the northeast-southwest direction and along the main stream of the Yellow River, with the center of distribution located at the junction of Liaocheng and Jinan.

#### 3.1.3. Spatial Proximity between WPIIs and Rivers

To further analyze the spatial relationship between WPIIs and rivers in the YRB, the number of enterprises within 10 km of the main stream of the Yellow River and its main tributaries were counted for WPIIs in 2013. The results are shown in Table 2, and the distribution of WPIIs in downstream areas and along important tributaries was dense. The number of WPIIs along the rivers in upstream areas was low. The large number of enterprises along the Fen and Wei rivers, which are important tributaries in the middle reaches of the river, reached 483 and 390, respectively. WPIIs were clustered along the rivers, and the water environment was under greater pressure. The water quality in the Taiyuan, Linfen and Yuncheng sections of the Fen River in Shanxi was poor V, with serious water pollution. The downstream area contains a concentration of population and production activities, and enterprises along the river were densely distributed, with 424 and 322 in Jindi River and Dawen River, respectively.

#### 3.1.4. Kernel Density Analysis

**Figure 5:**
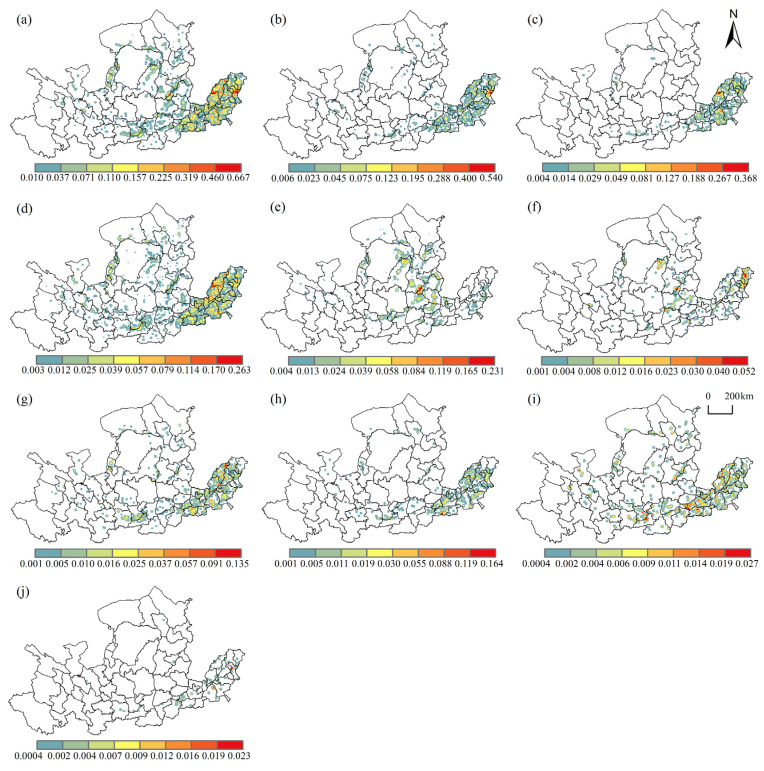
Kernel density of WPIIs in the Yellow River basin. (**a**) A total of 9 kinds of WPIIs; (**b**) manufacture of raw chemical materials and chemical products; (**c**) manufacture of textiles; (**d**) processing of food from agricultural products; (**e**) mining and washing of coal; (**f**) processing of petroleum, coking, processing of nuclear fuel; (**g**) manufacture of foods; (**h**) manufacture of paper and paper products; (**i**) manufacture of wine, drinks and refined tea; (**j**) manufacture of chemical fibers.

The kernel density analysis of 9 WPIIs in 2013 was carried out. The spatial distribution of all WPIIs in the YRB had significant watershed differentiation characteristics, with the intensity of downstream > midstream > upstream (Figure 5a). In the downstream, WPIIs were concentrated in high density, and enterprises were distributed in patches. In Zibo and Dezhou, high density cores were formed, and other cities show higher density polycentric patterns, respectively. In the midstream, Lvliang and Xi’an formed a high-density core, with enterprises forming a dense belt along the Fen and Wei rivers. In addition, in Ordos and Yulin, Yinchuan, Shizuishan and Baotou formed a beaded agglomeration zone along the main branches of the Yellow River.

There were industry differences in the spatial clustering of WPIIs (Figure 5b–j). Manufacture of raw chemical materials and chemical products, manufacture of chemical fibers, manufacture of paper and paper products, and manufacture of textiles were highly concentrated in the downstream area. Manufacture of raw chemical materials and chemical products had more toxic and harmful substances, with high pollution, high production value, high accident characteristics. It was mainly distributed in the downstream of the basin, forming a high-density core in the heavy industrial city of Zibo. The manufacture of textiles discharges a large amount of pollution, as it is a labor-intensive industry, with low value-added technological innovation and strong dependence on labor. It was concentrated in the labor-rich downstream, forming a high-density double core in the Liaocheng and Dezhou junction, Heze city. The effects of manufacture of paper and paper products and manufacture of chemical fibers on the water environment pollution was serious, and was mainly concentrated in the downstream areas.

Processing of food from agricultural products, manufacture of foods, manufacture of wine, drinks and refined tea mainly rely on the convenience of raw materials, and were distributed in the upstream, midstream and downstream, but the most dense distribution occurred in the downstream areas. Processing of food from agricultural products was the main contributor of NH_3_-N and COD, forming a number of high-density cores mainly in Dezhou and Liaocheng, with Lvliang and Xi’an as the cores; the enterprises formed two tandem clusters along Fen River and Wei River, and also formed a tandem cluster along the main stream of Yellow River from Yinchuan to Bayannur. Manufacture of foods formed a high-density core in Dezhou and Zhengzhou, with a small concentration near Xi’an. Manufacture of wine, drinks and refined tea formed a high-density core in Zhengzhou, Jiaozuo and Jiyuan, in addition to a small cluster near Xi’an.

Mining and washing of coal, processing of petroleum, coking, processing of nuclear fuel, as resource-intensive industries, are highly dependent on energy, so were mainly distributed in energy-rich regions. Shanxi, Shaanxi, and Inner Mongolia are the main supply bases of China’s energy resources, and mining and washing of coal was highly concentrated here. A high-density core was formed at the junction of Lvliang and Jinzhong, a cluster belt formed along the core to the periphery, and a small cluster belt is formed between Yulin and Ordos. Processing of petroleum, coking, processing of nuclear fuel were concentrated in the midstream and downstream, forming a high-density core in Dongying, an important oil base, and a small high-density core in Lvliang and Yulin, and it is connected to the periphery to form a dense belt.

### 3.2. Analysis of Influencing Factors

#### 3.2.1. Variables Selection

Drawing on relevant references, the spatial distribution characteristics of WPIIs, and taking into account the availability of data, this paper analyzes the factors influencing WPIIs from four aspects: resource endowment, socio-economic, pollution discharge, externality and transportation:

Resource endowment affects WPIIs mainly through natural resources, labor, and technology level [26,37]. Among them, natural resources are a basic factor for the production activities of the industry. Mining industries include resource-related industries such as coal, oil, gas and metals, and the abundance of resources can be reflected by the number of employees in mining industries. Labor affects WPIIs mainly through labor cost and labor quality, and the average wage of labor is used to reflect labor capital. Technological innovation can effectively promote industrial transformation and upgrading, improve resource utilization efficiency, and thereby reduce pollutant emissions, which is represented by science and technology expenditures.

Socio-economic factors are mainly characterized by the level of economic development and industrial structure. A better foundation of economic development, a more complete infrastructure and a large market capacity have an important impact on the industrial layout, represented by per capita GDP [38]. The regional specialization formed by the industrial historical base promotes the agglomeration of WPIIs, represented by the proportion of the secondary industry in GDP [20].

Pollution emission factors are characterized by the scale of industrial wastewater discharge and the distance from the WPIIs to the river. The scale of industrial wastewater discharge and the proximity of rivers have a direct impact on the discharge and treatment of wastewater, which are characterized by the industrial wastewater discharge and the nearest distance from the WPIIs to the river, respectively [39].

Externality and transportation factors are characterized by foreign investment and transportation. Foreign investment can provide various resource required by enterprises, thus affecting the spatial distribution pattern of WPIIs, which is represented by utilization of foreign capital [38]. The improvement of transportation conditions can remove the constraint of raw materials and enhance the accessibility of markets, which is represented by the location entropy of freight volume [40]. The selection and definition of various factors are shown in Table 3.

#### 3.2.2. Results of Regression Models

Since Ordinary Least Squares (OLS) is highly diagnostic in terms of factor covariance, etc., it is first analyzed by OLS, and the results are shown in Table 4. Taking the output of WPIIs in each region in 2013 as the dependent variable, and the selected 9 influencing factors as independent variables, the variance inflation factors (VIF) were all less than 7.5, indicating that there was no global multicollinearity between the factors. Average wage, externality and transportation factors did not pass the significance test at the 5% level. The number of employees in the mining industry, science and technology expenditure, per capita GDP, industrial structure, industrial wastewater discharge, and nearest distance to the river all passed the significance test at the 5% level. Therefore, the above six factors were further incorporated into the GWR model for analysis. We used GWR4 software, Gaussian kernel function model, and determined the optimal bandwidth by the golden section search method. The results of model parameters are shown in Table 5. The goodness of fit of the GWR model was 0.75, which was higher than the OLS model of 0.70, indicating that the GWR model fits better than the OLS model, and that each factor generates local regression coefficients for each study unit compared to the OLS model.

**Figure 6:**
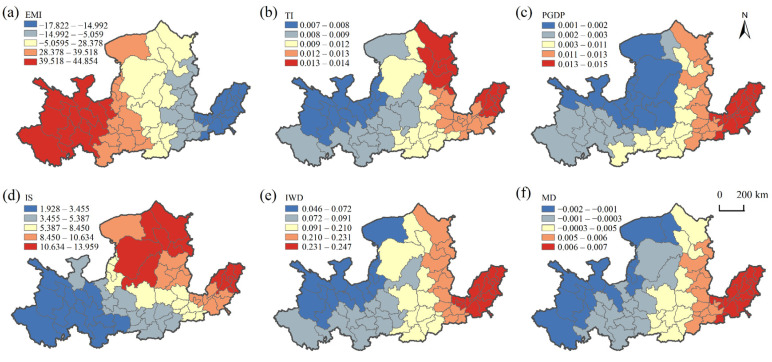
Spatial distribution of regression coefficients of GWR model. (**a**) Natural resource endowment; (**b**) technological innovation; (**c**) economic development level; (**d**) industrial structure; (**e**) industrial wastewater discharge scale; (**f**) minimum distance of WPIIs to rivers.

The influencing factors and the degree of role of WPIIs were usually different in different regions (Figure 6).

Resource endowment. The impact of natural resource endowments on the distribution of WPIIs gradually decreased from west to east, negatively (Figure 6a). The positive effect was greater in the central and eastern part of the midstream and upstream, indicating that natural resource endowment was the main factor promoting the distribution of WPIIs in the middle and upper reaches, and the layout of WPIIs in the middle and upper reaches was more restricted by the availability of raw materials. The Shanxi, Shaanxi, and Inner Mongolia regions are rich in energy resources, and WPIIs such as coal and oil that rely on energy are highly concentrated. The upstream area of Gansu, Qinghai and Ningxia has long been dependent on the development of agriculture and animal husbandry, and the distribution of processing of food from agricultural products and manufacture of foods there was dense. Different WPIIs depend on different types of resources, and their spatial distribution is thusly differentiated. The negative effect was mainly concentrated in the downstream, which had largely overcome the constraint of raw materials. The effect of technology innovation level on the WPIIs was significantly positive and mainly in the northeastern part of the basin, with a decreasing trend from Ulanqab, Xinzhou, Binzhou and Jinan to the southwest (Figure 6b). The eastern region has a better economic foundation and can better afford to invest in scientific research of WPIIs, while technological innovation promotes the transformation and upgrading of industries and improves the efficiency of resource utilization, thus promoting the agglomeration of WPIIs.

Socio-economic factors. The effect of economic development level on WPIIs was significantly positive, and the intensity of the effect decreased from east to west (Figure 6c). The stronger role of the region is located at the downstream and most of the midstream. This part of the economic foundation is better developed, the infrastructure is more complete, and the market scale and technology spillover can promote the development of WPIIs. The industrial structure dominated by secondary industry had a promoting effect on the development of WPIIs, and the degree of influence was high in the northeast and low in the southwest within the basin (Figure 6d). The regions with greater influence were Yulin, Erdos and Zibo, which are significantly influenced by the “industrial history foundation” and have a certain path dependence. For example, Baotou Iron and Steel, Yulin, Datong Coal, Zibo Chemical, Dongying Petroleum, Dezhou Textile, etc., attract WPIIs due to historical regional specialization and industrial backward and forward linkages.

Pollution discharge factors. The impact of industrial wastewater discharge scale on WPIIs was significantly positive, and the degree of impact decreased from east to west, indicating that the scale of pollution discharge is an important factor affecting the distribution of WPIIs in the middle and downstream (Figure 6e). Due to the proximity of the middle and downstream areas to river borders and sea inlets, the pollutants discharged can flow into the downstream areas through the carrying capacity of rivers and directly into the sea. In addition, the strong self-purification and discharge capacity of rivers had an important influence on the spatial layout of industries such as chemical industry and textile industry, which have a large discharge volume. The distance from the enterprise to the river has a decreasing impact on WPIIs from east to west, with the negative impact being mainly in the upstream, and the positive impact being concentrated in the middle and downstream areas (Figure 6f). Some areas in the upstream of the YRB were water-conserving areas with strong ecological constraints, which restrict the development of WPIIs. In the middle and downstream, the main stream of the Yellow River and the ocean have stronger pollution absorption capacity, the water environment is relatively relaxed, and most of the WPIIs are more dependent on water resources, therefore, WPIIs in the middle and downstream tend to be distributed along the river.

## 4. Discussion

This paper focuses on industrial point source pollution that has a serious impact on the water environment and water pollution events. The water pollution-intensive index was constructed by combining the scale-intensity method with industrial wastewater discharged (IWW), chemical oxygen demand discharged (COD), and ammonia nitrogen discharged (NH_3_-N) to quantitatively identify WPIIs. Comparing the first national pollution source census Bulletin (2010) [41], the results of WPIIs identified by the scale-intensity method are more realistic and accurate, and can effectively identify WPIIs and provide a quantitative identification method. 

WPIIs such as petrochemical industry, textile industry and agro-food processing industry in the YRB are mostly traditional industries which are facing the problems of low resource utilization, high consumption and high pollution. The concentration of WPIIs along the river has caused huge pressure on the water environment, resulting in some sections of the YRB approaching the upper limit of environmental capacity. The layout of WPIIs in the YRB is influenced by factors such as economic development, industrial structure and pollution discharge. These results verified the analysis of Zhou et al. [26]. Zhou et al. [39] emphasized the locational determinants of PIIs. The influencing factors and degree of effect in different regions was usually different. Our results further explain how different factors have positive or negative effects to varying degrees. It is worth noting that the development of WPIIs in the YRB is still highly dependent on resource endowments and is significantly affected by the historical foundation of the industry.

Therefore, it is of great value and significance to fully understand the location, scope and degree of agglomeration of WPIIs and their sub-industry in terms of spatial distribution and their influence mechanisms, to promote the transformation and upgrading of traditional industries, to promote the transfer of WPIIs, and to connect the ecological protection and high-quality development of the YRB, especially the protection of water resources, water environment and water ecology.

## 5. Conclusions

The scale of WPIIs in the YRB showed a growth trend from 2003 to 2013, with the output value increased from 442.5 billion yuan in 2003 to 6192.3 billion yuan in 2013, an increase of 13 times. Within the basin, the spatial distribution of industries is extremely uneven, showing a significant geographical differentiation of downstream > midstream > upstream. The WPIIs in the YRB showed east-west distribution characteristics, and the distribution center was basically located at the junction of the middle and lower reaches. The distribution of WPIIs in the upper, middle and lower reaches of the river basin was similar all along the northeast-southwest direction (along the direction of the river) and basically showed symmetrical distribution along the river. The spatial distribution of WPIIs was riverine and densely distributed in the downstream areas and along the important tributaries of the Fen and Wei rivers.

The WPIIs were clustered in high density in the downstream areas, and formed a cluster area centered on Zibo and Dezhou, as well as dense belts along the Fen River and Wei River. There were industrial differences in the spatial clustering of WPIIs. The chemical and textile industries were mainly concentrated in downstream areas. Processing of food from agricultural products was distributed in the upper, middle and lower reaches. Resource-intensive industries such as coal and oil were concentrated in the energy-rich midstream areas.

The spatial differences in the effects of each factor on the distribution of WPIIs were significant. Natural resource endowment was the main factor promoting the concentration of WPIIs in the middle and upstream of the basin, while the downstream had overcome resource constraints. The level of technological innovation had a significant impact on the eastern and downstream areas of the basin. The level of economic development and the historical foundation of industry promoted the geographical concentration of industry and formed a certain path dependence. The scale of wastewater discharge and the proximity of rivers also had a strong role in promoting the clustering of industries in the middle and downstream areas.

We offer some policy recommendations in this paper. (1) The local governments need to control the total amount of pollutants discharged according to the water environment capacity of the region. This can be implemented similarly to carbon emission trading, combined with the regional water environment capacity, implementation of the emission trading scheme, and reasonably laying out the transferred industries. (2) The implementation of environmental regulations should be adapted to the regional development stage and factor endowment. Environmental controls at river junctions should be appropriately strengthened to avoid transboundary pollution. (3) Industrial parks are an important means of industrial layout and development. Increasing the admission rate of WPIIs to the parks helps to centralize the layout of pollutant treatment facilities. At the same time, it promotes technological innovation and the transformation and upgrade of low-end industries, and encourages the development of high value-added and technology-intensive industries.

## Figures and Tables

**Table 1:** Water pollution-intensive index of industries.

Industry Code	Industry	*I*
22	Manufacture of paper and paper products	0.78
26	Manufacture of raw chemical materials and chemical products	0.57
17	Manufacture of textiles	0.37
13	Processing of food from agricultural products	0.35
28	Manufacture of chemical fibers	0.33
15	Manufacture of wine, drinks and refined tea	0.32
06	Mining and washing of coal	0.29
25	Processing of petroleum, coking, processing of nuclear fuel	0.24
14	Manufacture of foods	0.22

**Table 2:** Spatial relationship between WPIIs and rivers in 2013.

Main Tributaries	Number of WPIIs	Main Tributaries	Number of WPIIs
Main stream of the Yellow River	1970	Wuding River	25
Huangshui River	73	Fen River	483
Datong River	13	Wei River	390
Tao River	11	Beiluo River	42
Zuli River	16	Yiluo River	193
Qingshui River	32	Qin River	183
Dahei River	54	Jindi River	424
Kuye River	114	Dawen River	322

**Table 3:** Description of influencing factors.

Category	Factors	Definition	Abbreviation	MEAN	MAX	MIN	SD
Resources endowment	Labor capital	Average wage of labor (yuan)	LC	45,446	256,877	8407	40,397
	Natural resource endowment	Number of employees in the mining industry (10,000 people)	EMI	3.8485	20.72	0.0008	4.8529
	Technological innovation	Science and technology expenditure (10,000 yuan)	TI	28,120	172,649	409	30,980
Socio-economic	Economic development level	Per capita GDP (Yuan)	PGDP	46,927	256,877	8407	40,397
	Industrial structure	Proportion of secondary industry (%)	IS	52.95	74.78	25.60	11.77
Pollutant discharge	Industrial wastewater discharge scale	Industrial wastewater discharge (10,000 t)	IWD	4708	15,921	26	4073
	River proximity	Minimum distance of WPIIs to rivers (km)	MD	29,388	105,466	41	27,707
Externality and transportation	Foreign investment	Utilization of foreign capital (USD 10,000)	FI	37,919	332,178	0	65,875
	Transportation	Location quotient index of the amount of freight traffic (%)	TRAN	7.73	42.34	0.13	7.19

**Table 4:** Coefficient estimation of OLS model.

Factors	Coefficient Estimation	Standard Deviation	VIF
LC	0.002	0.015	2.107
EMI	−3.877 *	20.102	1.643
TI	0.011 *	0.005	3.881
PGDP	0.011 **	0.003	2.766
IS	3.646 *	7.550	1.364
IWD	1.364 **	0.029	2.391
MD	0.003 *	0.003	1.265
FI	−0.009	0.002	3.704
TRAN	0.005	1.141	3.363
R^2^	0.702		

** represents *p*-value significant at 1% level, * represents *p*-value significant at 5% level.

**Table 5:** Coefficient estimation of GWR model.

Serial No.	Coefficient	Value
1	Best bandwidth size	64.00
2	Residual sum of squares	14,765,541.49
3	−2 log-likelihood	1000.29
4	Classic AIC	1034.72
5	AICc	1047.83
6	BIC/MDL	1072.40
7	CV	501,133.71
8	R square	0.83
9	Adjusted R square	0.75

## Data Availability

The data presented in this study are available on request from the author. The data are not publicly available due to privacy.

## References

[1] Kahn M.E., Li P., Zhao D. (2015). Water pollution progress at borders: The role of changes in China’s political promotion incentives. Am. Econ. J. Econ. Policy..

[2] Li P. (2020). To Make the Water Safer. Expo. Health.

[3] Huang Y., Sui Q., Lyu S., Wang J., Huang S., Zhao W., Wang B., Xu D., Kong M., Zhang Y. (2020). Tracking emission sources of PAHs in a region with pollution-intensive industries, Taihu Basin: From potential pollution sources to surface water. Environ. Pollut..

[4] Lu D., Sun D. (2019). Development and management tasks of the Yellow River Basin: A preliminary understanding and suggestion. Acta Geogr. Sin..

[5] Hu J., Liu Y., Fang J., Jing Y., Liu Y., Liu Y. (2019). Characterizing pollution-intensive industry transfers in China from 2007 to 2016 using land use data. J. Clean. Prod..

[6] Chen Y., Fu B., Zhao Y., Wang K., Zhao M., Ma J., Wu J., Xu C., Liu W., Wang H. (2020). Sustainable development in the Yellow River Basin: Issues and strategies. J. Clean. Prod..

[7] China Ecological Environment Status Bulletin 2018. https://www.mee.gov.cn/hjzl/sthjzk/zghjzkgb/.

[8] Zhao H., Liu Y., Lindley S., Meng F., Niu M. (2020). Change, mechanism, and response of pollutant discharge pattern resulting from manufacturing industrial transfer: A case study of the Pan-Yangtze River Delta, China. J. Clean. Prod..

[9] Han D., Huang G., Liu L., Zhai M., Gao S. (2021). Multi-regional industrial wastewater metabolism analysis for the Yangtze River Economic Belt, China. Environ. Pollut..

[10] Wang Q., Yang Z. (2016). Industrial water pollution, water environment treatment, and health risks in China. Environ. Pollut..

[11] Cole M.A. (2004). Trade, the pollution haven hypothesis and the environmental Kuznets curve: Examining the linkages. Energy Econ..

[12] Yuan F., Wei Y.D., Gao J., Chen W. (2019). Water crisis, environmental regulations and location dynamics of pollution-intensive industries in China: A study of the Taihu Lake watershed. J. Clean. Prod..

[13] Fu S., Ma Z., Ni B., Peng J., Zhang L., Fu Q. (2021). Research on the spatial differences of pollution-intensive industry transfer under the environmental regulation in China. Ecol. Indic..

[14] Wu Y., Yu S., Duan X. (2021). The Impact of Environmental Regulation on the Location of Pollution-Intensive Industries in China under Agglomeration Effect. Int. J. Environ. Res. Public Health.

[15] She Y., Liu Y., Deng Y., Jiang L. (2020). Can China’s government-oriented environmental regulation reduce water pollution? Evidence from water pollution intensive firms. Sustainability.

[16] Chang Y. (2017). Types identification for regional water pollution-intensive manufacturing industry and development path: Case of water pollution-intensive manufacturing industry in Jiangsu. Yangtze River.

[17] Ji X., Song Y., Sun Y., Wang D., Meng H., Li S., Huang X. (2020). Spatial-temporal variation of chemical industry and its influencing factors in the Yangtze River Delta from the perspective of industrial park admission rate of chemical enterprises. Geogr. Res..

[18] Chen L., Caro F., Corbett C.J., Ding X. (2019). Estimating the environmental and economic impacts of widespread adoption of potential technology solutions to reduce water use and pollution: Application to China’s textile industry. Environ. Impact Assess. Rev..

[19] Zhang C., Chen J., Wen Z. (2012). Alternative policy assessment for water pollution control in China’s pulp and paper industry. Resour. Conserv. Recycl..

[20] He C., Zhu S., Wang J., Pan F. (2010). The Location of China’s Manufacturing Industry.

[21] Grether J.M., Hotz I., Mathys N.A. (2014). Industry location in Chinese provinces: Does energy abundance matter?. Energy Econ..

[22] He C., Pan F., Chen T. (2016). Research progress of industrial geography in China. J. Geogr. Sci..

[23] Zhang G., Liu W., Duan H. (2020). Environmental regulation policies, local government enforcement and pollution-intensive industry transfer in China. Comput. Ind. Eng..

[24] Shen J., Wang S., Liu W., Chu J. (2019). Does migration of pollution-intensive industries impact environmental efficiency? Evidence supporting “Pollution Haven Hypothesis”. J. Environ. Manag..

[25] Ramanathan R., He Q., Black A., Ghobadian A., Gallear D. (2017). Environmental regulations, innovation and firm performance: A revisit of the Porter hypothesis. J. Clean. Prod..

[26] Zhou Y., He C., Liu Y. (2015). An Empirical Study on the Geographical Distribution of Pollution-Intensive Industries in China. J. Nat. Resour..

[27] Zhou Y., Zhu S., He C. (2017). How do environmental regulations affect industrial dynamics? Evidence from China’s pollution-intensive industries. Habitat Int..

[28] Sigman H. (2005). Transboundary spillovers and decentralization of environmental policies. J. Environ. Econ. Manag..

[29] Huang X., He P., Zhang W. (2016). A cooperative differential game of transboundary industrial pollution between two regions. J. Clean. Prod..

[30] China City Statistical Yearbook (2019). China’s Economic and Social Big Data Research Platform. https://data.cnki.net/Yearbook/Single/N2020050229.

[31] Xi J. Speech at the Symposium on Ecological Protection and High-Quality Development in the Yellow River Basin. Seeking Truth. http://www.qstheory.cn/dukan/qs/2019-10/15/c_1125102357.htm.

[32] Liu Q., Wang Q., Li P. (2012). Regional Distribution Changes of Pollution-Intensive Industries in China. Ecol. Econ..

[33] Qiu F., Jiang T., Zhang C., Shan Y. (2013). Spatial Relocation and Mechanism of Pollution-intensive Industries in Jiangsu Province. Sci. Geogr. Sin..

[34] Shi Y., Matsunaga T., Yamaguchi Y., Li Z., Gu X., Chen X. (2018). Long-term trends and spatial patterns of satellite-retrieved PM2.5 concentrations in South and Southeast Asia from 1999 to 2014. Sci. Total Environ..

[35] Wang J., Liao Y., Liu X. (2019). Tutorial on Spatial Data Analysis.

[36] Wang S., Shi C., Fang C., Feng K. (2019). Examining the spatial variations of determinants of energy-related CO_2_ emissions in China at the city level using Geographically Weighted Regression Model. Appl. Energy.

[37] Ren M., Huang C., Wang X., Hu W., Zhang W. (2019). Research on the distribution of pollution-intensive industries and their spatial effects in China. Sustainability.

[38] Song Z., Liu W. (2013). Spatial distribution of small and medium-sized enterprises (SMEs) and its determinants in China. Geogr. Res..

[39] Zhou M., Tan S., Guo M., Zhang L. (2015). Locational determinants of emissions from pollution-intensive firms in urban areas. PLoS ONE.

[40] Wu J., Wei Y.D., Chen W., Yuan F. (2019). Environmental regulations and redistribution of polluting industries in transitional China: Understanding regional and industrial differences. J. Clean. Prod..

[41] The First National Pollution Source Census Bulletin. http://www.stats.gov.cn/tjsj/tjgb/qttjgb/qgqttjgb/201002/t20100211_30641.html.

